# Psychometric Properties of the Caring Behaviors Inventory-16 in Ethiopia

**DOI:** 10.3390/nursrep12020037

**Published:** 2022-05-26

**Authors:** Abebaw Jember Ferede, Kerstin Erlandsson, Lemma Derseh Gezie, Biftu Geda, Lena Wettergren

**Affiliations:** 1 Department of Medical Nursing, School of Nursing, College of Medicine and Health Sciences, University of Gondar, Gondar 196, Ethiopia; 2 School of Education, Health and Social Studies, Dalarna University, 791 88 Falun, Sweden; ker@du.se; 3 Department of Women’s and Children’s Health, Division of Reproductive Health, Karolinska Institute, 171 77 Stockholm, Sweden; lena.wettergren@pubcare.uu.se; 4 Department of Epidemiology and Biostatistics, Institute of Public Health, College of Medicine and Health Sciences, University of Gondar, Gondar 196, Ethiopia; lemmagezie@gmail.com; 5 Department of Nursing, College of Health Science, Shashamene Campus, Madda Walabu University, Robe 247, Ethiopia; biftug@gmail.com; 6 Department of Public Health and Caring Sciences, Uppsala University, 751 22 Uppsala, Sweden

**Keywords:** Amharic, nursing care, caring behaviors inventory-16, cultural adaptation, Ethiopia, nurse caring behaviors, patient care, psychometrics, translation

## Abstract

*Background:* The Caring Behaviors Inventory-16 (CBI-16) is a comprehensive instrument measuring caring behaviors as experienced by patients. The study aimed to translate, culturally adapt and evaluate the psychometric properties of the CBI-16 among adult patients who speak the Amharic language. *Methods:* The measure was completed by 304 hospitalized patients. Construct validity was evaluated via exploratory factor analysis (EFA), confirmatory factor analysis (CFA), and contrasted groups’ validity. Total CBI-16 scores were compared between groups that differed in self-rated satisfaction with care (Patient Satisfaction Instrument) to examine the contrasted groups’ validity. Reliability was assessed using internal consistency (Cronbach’s alpha). *Results:* The EFA suggested a four-factor model accounting for 66.1% of the total variance. The items loaded onto the subscales were similar to the CBI-24. The CFA supported the four-factor model with acceptable fit indices: normed Chi-square value 2.65 (*X*^2^ = 259.60, df = 98), SRMR = 0.06, and RMSEA = 0.07, CFI = 0.88 and TLI = 0.86. The contrasted groups’ validity was supported by significantly higher CBI-16 scores reported by patients more satisfied with their care (t = 3.66, *p* < 0.001). The reliability of the instrument was satisfactory (Cronbach’s alpha = 0.83). *Conclusions:* The Amharic version of the CBI-16 displayed a four-factor solution and was shown to be a valid and reliable instrument for the assessment of the perceptions of caring behaviors in Ethiopia.

## 1. Background

Caring is a complex and interactive process that occurs between a nurse and a patient in a professional interpersonal relation [1]. The literature describes two main aspects of caring, expressive and instrumental, which constitute nurses’ behaviors when responding to patients’ care needs. Expressive caring behaviors involve providing psychological support, and instrumental caring behaviors are physical-oriented activities that promote comfort and coping. Nurses’ caring behaviors are directed toward the welfare of the patient [2] and are significant for clinical practice [3]. However, exploring dimensions of patient care can be challenging because caring is a complex phenomenon [4].

Understanding caring behaviors presents opportunities for nurses to have knowledge about how patients perceive the care they provide and how patients prefer to be cared for. The measurement of caring behaviors could therefore be the basis for the improvement of practices and outcomes of care [5,6]. One of the most commonly used instruments to measure caring behaviors is the Caring Behaviors Inventory (CBI) [7]. The measure was developed by Zane Robinson Wolf based on Jean Watson’s theory of human caring, the most inclusive and widely used model of caring [1,7].

The CBI was developed based on a clear conceptual-theoretical basis and has been psychometrically tested and refined in an ongoing way [5,8]. It was originally developed as a 75-item scale and then reduced to a 43-item (five subscales) and, finally, to a 42-item scale [7]. The tool was thereafter revised through psychometric testing to a 24-item scale (four subscales) [5] and further reduced to a unidimensional 16-item scale [8]. The last version of the instrument has been translated into two other languages, of which one revealed a two-factor component [9] indicating that the 16-item version requires further psychometric evaluation, especially in cultures different from the US. The CBI has been used to determine perceptions of nurse caring behaviors among patients [10,11,12,13], nurses [14,15], patients’ caregivers [16,17] and students [18,19] and to compare perceptions of caring behaviors between nurses and patients [20,21].

Translating and evaluating the psychometric properties of an instrument in a new setting (in terms of culture and language) is important to ensure that the concepts being measured are correctly captured. Furthermore, the information regarding patients’ and nurses’ perceptions of caring behaviors in non-English-speaking countries, especially in African countries, is limited [22]. Thus, the aim of this study was to translate, culturally adapt and evaluate the psychometric properties of the CBI-16 in the Amharic language among adult medical and surgical patients in Ethiopia.

## 2. Methods and Materials

### 2.1. The Caring Behaviors Inventory-16

The CBI-16 is a 16-item unidimensional instrument. Responses are provided on a six-point Likert-type scale (1 = never to 6 = always). The total score ranges from 16 to 96, where higher values reflect better perceptions of the caring behaviors nurses carry out [8]. The CBI-16 shows high internal consistency (Cronbach’s alpha = 0.95).

### 2.2. Translation and Cultural Adaptation of the CBI-16

The translation process was conducted in accordance with the guidelines by Beaton et al. [23]. First, the instrument was translated into Amharic by two independent bilingual translators: a healthcare professional who was informed about the underlying concept of the items and a language expert who was blinded. Second, the forward translations were reviewed by both translators, and a synthesized forward-translated version was established. Third, the translated version was back-translated by two independent bilingual translators who were not aware of the intentions behind the items. Fourth, the back-translations were reviewed and synthesized by both back-translators, who then reached a synthesized back-translated version. Next, an expert review committee, moderated by the primary investigator and composed of the forward- and back-translators and a nurse researcher, evaluated and compared the translations and reached an Ethiopian Amharic prefinal version of the CBI-16 (Table S1) [23]. Lastly, cognitive interviews were performed with 15 adult hospitalized patients aged 18–65 years who were admitted to internal medicine and surgery units for at least two days. Respondents were purposively selected by considering sex, age, residence and educational status [23]. Based on the interviews, some translated items underwent minor modifications after thorough discussions in the review committee. More than half of the respondents replied that one of the items (item 8: Demonstrating professional knowledge and skill.) was difficult to complete because they did not know if their nurses were knowledgeable or not. Similarly, item 10 (Treating your information confidentially.) was unclear for eight participants and was therefore rephrased in Amharic as ‘Does the nurse keep your information confidential?’.

### 2.3. Design and Setting

A cross-sectional study was conducted at three referral hospitals situated in the Amharic-speaking region of Ethiopia: University of Gondar Comprehensive Specialized Hospital (UOGCSH), Debre Markos Referral Hospital and Debre Birhan Referral Hospital. Data were collected during the 2-month period between December 2020 and February 2021.

### 2.4. Participants

Adult patients aged 18–65 years who were admitted to internal medicine and surgery units for at least two days (in order to have received nursing care and be able to judge it) were approached regarding participation. The sample size was based on the recommendations by Harrington of at least 300 respondents to conduct CFA [24]. Therefore, all 308 adult patients admitted to the internal medicine and surgery units of the three referral hospitals during the data collection period were consecutively enrolled in the study. Patients who had known cognitive impairment or other communication barriers were excluded.

### 2.5. Data Collection

All patients were asked to respond to a survey package which included the CBI-16, the Patient Satisfaction Instrument (PSI) and questions on socio-demographic (marital status, residence and education) and clinical characteristics (hospitalization days, number of admissions in the last five years and time spent with the nurse in minutes).

### 2.6. Patient Satisfaction Instrument

The 25-item PSI was used to measure patients’ satisfaction with the received nursing care [25]. Responses are presented on a five-point Likert-type scale (ranging from strongly disagree = 1 to strongly agree = 5). Total response scores range from 1 to 5, with higher scores indicating higher levels of satisfaction. The PSI showed high internal consistency (Cronbach’s alpha = 0.94) [26]. The PSI was translated into Amharic via standard forward- and back-translation procedures [23].

### 2.7. Data Collection Procedure

Data were collected by two nurses with experience in collecting survey data. The nurses, who did not work in the internal medicine and surgery units at the referral hospitals, performed face-to-face interviews using a structured printed questionnaire. The average time to complete the interviews was 25 min.

### 2.8. Data Quality Control

Data quality was ensured by training the data collectors, coding the questionnaires before data collection and cross-checking for the consistency and completeness of the return questionnaires every day. Floor and ceiling effects were calculated and considered acceptable if they did not exceed 15% [27].

### 2.9. Statistical Analysis

Stata-16 was used for statistical analysis. Demographic characteristics are presented using frequencies, percentages, means and standard deviations (SDs). The Shapiro–Francia W test of normality indicated that some items on the CBI-16 Ethiopian Amharic version had a normal distribution and others moderately deviated from the normal distribution. Therefore, a maximum likelihood estimator with a Satorra–Bentler scale was used [24,28].

### 2.10. Construct Validity

Data factorability was checked using a Kaiser–Meyer–Olkin (KMO) test and a Bartlett test of sphericity. KMO values between 0.8 and 1.0 were considered good, and a significant Bartlett test of sphericity (*p <* 0.05) indicated sampling adequacy for factor analysis. Then, EFA was performed using principal-component factors; eigenvalues ≥ 1 and the scree plot of these eigenvalues were used to identify the components to be retained. The components were rotated using Orthogonal Varimax rotation with Kaiser normalization, and variables with loadings of ≥0.4 were retained in the model.

Confirmatory factor analysis was conducted using the Satorra–Bentler maximum likelihood estimator to examine the factor structure of the CBI-16 Ethiopian Amharic version revealed in the EFA. To judge model fit, a normed Chi-square test (*X*^2^/df) value of <3 was considered an acceptable fit [29], a comparative fit index (CFI) > 0.90 and a Tucker–Lewis Index (TLI) > 0.90 were considered a good fit. A root mean square error of approximation (RMSEA) ≤ 0.08, and a standard root mean square residual (SRMR < 0.08) were considered an acceptable fit [28,30]. The contrasted groups’ validity was examined by comparing patients divided into two groups based on the level of satisfaction. An independent samples *t*-test was used to compare total CBI-16 scores between those reporting high satisfaction and those reporting low satisfaction [29].

### 2.11. Reliability

Internal consistency reliability was estimated for the total CBI-16 Ethiopian Amharic version using Cronbach’s alpha coefficient. The following were evaluated: Cronbach’s alpha values, for which values between 0.70 and 0.90 were considered good; the item-test correlation, which should be roughly the same for all items; item-rest correlation; the average inter-item correlation, which should be between 0.20 and 0.40, and the Cronbach’s alpha value if an item was deleted [31].

## 3. Results

A total of 304 questionnaires were included in the analysis, yielding a 98.7% response rate. The mean (SD) age of the sample was 36.5 (13.4) years; male respondents accounted for 61.8% of the sample. Most of the respondents were married (59.9%), about half had urban residency (52.3%) and (52.6%) were admitted for medical conditions and being cared for in internal medicine units (Table 1).

The total mean (SD) of the CBI-16 Ethiopian Amharic version and patients’ satisfaction levels were 3.74 (0.43) and 3.58 (0.40), respectively. All response alternatives in the six-point Likert scale were used for all items. Floor and ceiling effects did not exceed 5.0%. Descriptive statistics for items and subscales are presented in Table 2.

### 3.1. Construct Validity

**Figure 1:**
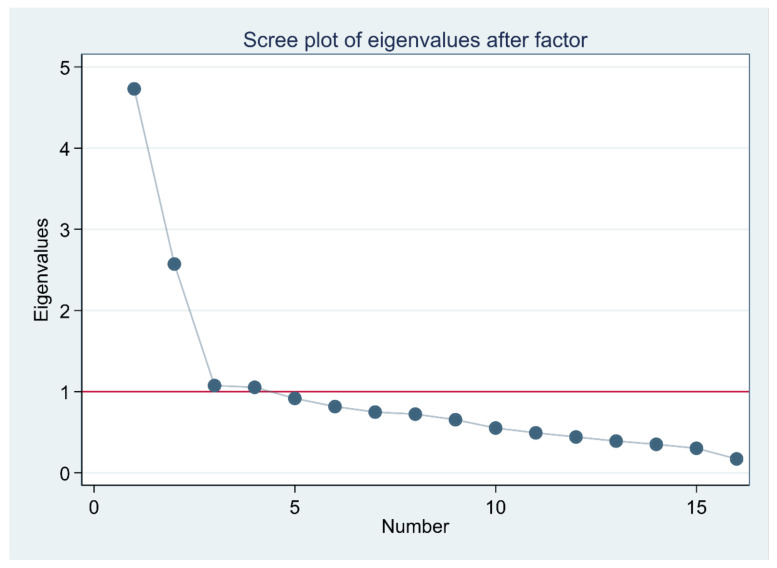
Scree plot of the eigenvalues for the Caring Behaviors Inventory–16.

The value of the KMO test (0.80) for sampling adequacy and the Bartlett test of sphericity (*X*^2^ = 1707.78, *p* < 0.001) showed sampling adequacy. The principal-component factors revealed a four-component solution explaining 66.1% of the total variance, as presented in the scree plot (Figure 1). One item (item 13: Meeting your stated and unstated needs.) loaded to the ‘knowledge and skill’ subscale instead of the ‘respectful’ subscale (Table 2).

**Figure 2:**
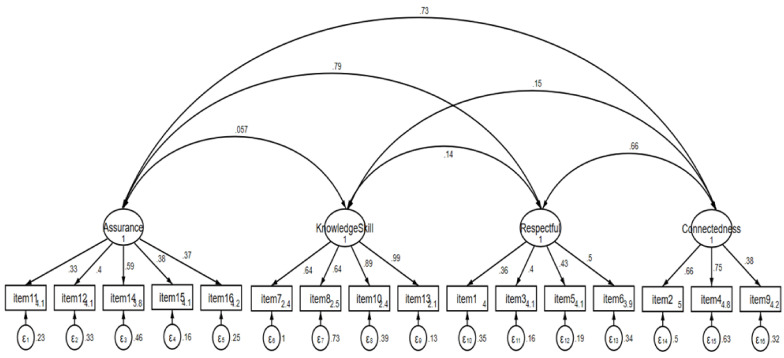
Confirmatory Factor Analysis Model Standardized Coefficients of the Caring Behaviors Inventory-16.

The CFA showed acceptable model fit, with a normed Chi-square value of 2.65 (*X*^2^ = 259.602, df = 98, *p* < 0.001), SRMR = 0.06 and RMSEA = 0.074 (90% CI 0.074–0.095, *p* < 0.01). The CFI = 0.88 and TLI = 0.86 indicated values close to 0.90, reflecting acceptable fit. Contrasted groups validity showed that groups differed by satisfaction level (t = 3.66, *p* < 0.001); total CBI-16 scores differed by satisfaction level (high satisfaction M(SD) = 3.83 (0.45) versus low satisfaction M(SD) = 3.66 (0.40)). The overall equation-level goodness of fit showed an *R^2^*-value of 0.99. Most of the item reliabilities (*R^2^*-values) of the CBI-16 Ethiopian Amharic version were higher than 0.40 and therefore acceptable. However, some items had values of 0.36 or less. The lowest item reliability with *R^2^* = 0.27 was found for item-1 (Table 2). The CFA model is presented in Figure 2.

### 3.2. Reliability

The overall Cronbach’s alpha coefficient for the CBI-16 Ethiopian Amharic version was 0.83, demonstrating satisfactory internal consistency reliability. The subscales also showed good internal consistency reliability for Assurance (0.75), Knowledge and Skill (0.82) and Respectful (0.74). The Connectedness subscale had the lowest value (0.67) among the subscales. The item-test correlation coefficients ranged from 0.40 to 0.63, and the item-rest correlation coefficients ranged from 0.29 to 0.56. Some of the items had values between 0.29 and 0.35, whereas item 8 and item 13 had the lowest (0.29) (Table 2). The average inter-item correlation was 0.23.

## 4. Discussion

Reliability and validity are essential characteristics of any measurement tool [29]. In this study, the CBI-16 was translated into Amharic, culturally adapted, and psychometrically examined with regard to construct validity and reliability. Conceptual equivalence was pursued during translation proceedings and through cognitive interviewing [23]. This is the third time the CBI-16 has been psychometrically evaluated following Alikari et al. [6] and Ghafouri et al. [9].

According to the EFA results, the 16 items loaded on four factors based on eigenvalue and the scree plot considerations [28], explaining 66.1% of the total variance. The four-factor model revealed in this study is in line with the factor structure of the CBI-24 (Assurance, Knowledge and Skill, Respectful, and Connectedness) [5], though one item (item 13) loaded differently. However, this contrasts with Wolf et al. [8] and Alikari et al. [6] who both supported a one-component solution, and Ghafouri et al., which found a two-factor component [9]. The difference in factor structure across studies needs to be further investigated. At this point, we do not know if our factor solution is related to the delivery of care in healthcare settings in a low-income country or other factors. We recommend the concept to be further studied, for example, by interviewing patients and nurses about care.

The CFA model was fitted after EFA. The overall model fit was demonstrated with a normed Chi-square, RMSEA and SRMR, which showed an acceptable fit, and the CFI and TLI produced values close to 0.90, which also reflect an acceptable fit. The related literature indicates that more than one model-fit index should be reported [28]. The structural validity values indicated that the items of the four subscales converge so that they measure the underlying constructs of the latent variables. The CBI-16 Ethiopian Amharic version showed evidence of contrasted groups’ validity because perceptions of nurses’ caring behaviors differed between groups with high and low levels of satisfaction regarding received care. The contrasted groups’ validity was reported by Wolf et al. in that total CBI-16 scores differed based on self-rated health status [8].

The internal consistency reliability of the total score, as well as the reliabilities of subscales, were satisfactory, with the exception of the Connectedness subscale, which was slightly below the recommended alpha of 0.70 but still acceptable [32]. Most of the item loadings were lower than those in studies by Wolf et al. [8] and Alikari et al. [6]. The Knowledge and Skill subscale showed the lowest loadings. This could be explained by the difference in cultural background between the patients in this study and those in previous studies [6].

The item reliability tests showed satisfactory results. The item-test correlations showed roughly the same results for all items. Regarding the item-rest correlations, some of the items showed low values, especially item 8 and item 13. When these items are deleted, the Cronbach’s alpha value did not substantially decrease, nor did the average inter-item correlation substantially increase. If the items were not important, these values would be expected to either substantially increase or decrease. Therefore, the items were retained in the analysis. This finding indicates the stability and reliability of the scale. The low item reliability test values may be a result of the interview-based administration of the instrument, suggesting that the CBI-16 Ethiopian Amharic version may benefit from being a self-report instrument.

Overall, the measures indicate that the CBI-16 Ethiopian Amharic version is an acceptable and ready-to-use research instrument in Amharic-speaking populations in Ethiopian healthcare settings. It can be translated into other languages in the country to measure perceptions of caring behaviors.

### Strengths and Limitations

This study is a report based on the actual perceptions of nurse caring behaviors while patients were being admitted. The high response rate and large sample size of 304 respondents enabled rigorous statistical analysis to evaluate validity.

There are a few limitations to be considered. The CBI-16 Ethiopian Amharic version was administered through interviews. The six-point Likert-type responses may have been affected by unease during data collection because the response options were difficult for the respondents to remember. During the use of the instrument in interview-based research, using a card with the response options written on it or conducting further studies concerning the reduction in the response options into five-point or other appropriate scale types may ease the interview process. Because the respondents were admitted to units during the period of data collection, the presence of other people in the units (nurses, doctors, patients’ caregivers and others) may have caused social desirability bias and affected the responses.

## 5. Conclusions

The CBI-16 Ethiopian Amharic version appears to be a valid and reliable instrument to assess perceptions of caring behaviors in four subscales. The instrument is recommended for use in research and clinical practice in Ethiopia. A systematic approach comprising translation, cultural adaptation and psychometric testing demands subsequent decisions.

## Figures and Tables

**Table 1:** Characteristics of participants in the psychometric evaluation of the CBI-16 (*n* = 304).

Characteristic	Median (Range)	*n* (%)
Sex		
Male		188 (61.8)
Female		116 (38.2)
Marital status		
Married		182 (59.9)
Single		108 (35.5)
Divorced		14 (4.6)
Residence		
Urban		159 (52.3)
Rural		145 (47.7)
Educational level		
No formal education		111 (36.5)
Can read and write		24 (7.9)
Elementary school		55 (18.1)
High school		64 (21.1)
Diploma		34 (11.2)
Bachelor and above		16 (5.3)
Current unit		
Internal medicine		160 (52.6)
Surgery		144 (47.4)
Hospitalization days	Median = 5 (2–210)	
Number of admissions in the last 5 years	Median = 1 (1–10)	
Time spent by the nurse in minutes	Median = 30 (5–480)	

CBI-16: Caring Behaviors Inventory-16

**Table 2:** Descriptive statistics of the Caring Behaviors Inventory-16 (*n* = 304).

Subscales	Mean (SD)	Correlation		α If Item Deleted	*R^2^*	Floor-Ceiling Effect
		Item-Test	Item-Rest			
**Assurance**	**4.07 (0.14)**					4.0–4.5
Item 11	4.09 (0.58)	0.55	0.46	0.82	0.31	4.0–4.5
Item 12	4.12 (0.70)	0.55	0.46	0.82	0.33	4.5–5.0
Item 14	3.82 (0.89)	0.60	0.52	0.82	0.43	4.0–4.5
Item 15	4.13 (0.56)	0.61	0.53	0.82	0.47	4.5–5.0
Item 16	4.17 (0.63)	0.54	0.45	0.82	0.36	4.0–4.5
**Knowledge and Skill**	**2.37 (0.17)**					2.0–2.5
Item 7	2.42 (1.19)	0.43	0.33	0.83	0.29	3.5–4.0
Item 8	2.55 (1.07)	0.40	0.29	0.83	0.36	2.0–2.5
Item 10	2.36 (1.08)	0.40	0.30	0.83	0.67	2.5–3.0
Item 13	2.14 (1.06)	0.40	0.29	0.83	0.88	4.0–4.5
**Respectful**	**4.03 (0.08)**					4.0–4.5
Item 1	4.01 (0.69)	0.46	0.36	0.83	0.27	2.0–2.5
Item 3	4.10 (0.56)	0.63	0.56	0.81	0.50	4.0–4.5
Item 5	4.08 (0.61)	0.60	0.52	0.82	0.49	4.0–4.5
Item 6	3.92 (0.77)	0.63	0.56	0.81	0.42	2.0–2.5
**Connectedness**	**4.66 (0.43)**					4.5–5.0
Item 2	4.99 (0.97)	0.56	0.47	0.82	0.47	3.5–4.0
Item 4	4.83 (1.09)	0.57	0.48	0.82	0.47	4.0–4.5
Item 9	4.17 (0.68)	0.57	0.46	0.82	0.32	4.0–4.5
**Overall**				**0.83**	**0.99**	

*R^2^*: Item reliability value, SD: Standard deviation.

## Data Availability

The datasets generated and analyzed for this study are available from the corresponding author upon reasonable request.

## Supplementary Materials

The following are available online at https://www.mdpi.com/article/10.3390/nursrep12020037/s1, Table S1: Items and responses of the CBI-16: Original vs. Ethiopian Amharic version.

## Abbreviations

CBI—Caring Behaviors Inventory, CFA—Confirmatory Factor Analysis, CFI—Comparative Fit Index, EFA—Exploratory Factor Analysis, KMO—Kaiser–Meyer–Olkin, PSI—Patient Satisfaction Instrument, RMSEA—Root Mean Square Error of Approximation, SD—Standard Deviation, SRMR—Standard Root Mean Square Residual, TLI—Tucker–Lewis Index, UOGCSH—University of Gondar Comprehensive Specialized Hospital.
