# Supplementary material for: Psychometric Properties of the Caring Behaviors Inventory-16 in Ethiopia
Source: Nurs Rep. 2022 May 26;12(2):387–96. doi: 10.3390/nursrep12020037 (PMC9229844; doi:10.3390/nursrep12020037)
Supplement: Supplementary file 1 [file nursrep-12-00037-s001.zip › nursrep-1723556-supplementary.pdf]

nursing reports

Supplementary Material

Table S1. Items and responses of the CBI-16: Original vs Ethiopian Amharic version

<table>
<tr><td colspan="6">The Caring Behaviors Inventory-16
የነርሶች እንክብካቤ ባህሪያት መጠይቅ-16</td></tr>
<tr><td colspan="6">Directions: Please read the list of items that describe nurse caring. For each item, please circle the respondent's response.
መመሪያ፡ እባክዎ የተዘረዘሩትን የነርስ እንክብካቤ መገለጫዎች በጥሞና ያንብቡ። ለእያንዳንዱ ጥያቄ ታካሚዎች የሚሰጡትን ምላሽ ያክብቡ።
Remember: You collect data from patients.
ያስታውሱ፡ መረጃ የሚሰበስቡት ከታካሚዎች ነው።</td></tr>
<tr><td colspan="6">1. Attentively listening to you.
1. ነርሷ/ነርሱ በጥሞና ያዳምጡዎታል?</td></tr>
<tr><td>Never
በጭራሽ</td><td>Almost never
ብዙ አያዳምጡም</td><td>Occasionally
አልፎ አልፎ</td><td>Usually
ብዙውን ጊዜ</td><td>Almost always
ሁልጊዜ ማለት ይቻላል</td><td>Always
ሁልጊዜ</td></tr>
<tr><td colspan="6">2. Giving instructions or teaching you.
2. ነርሷ/ነርሱ መመሪያ ወይም ትምህርት ይሰጡዎታል?</td></tr>
<tr><td>Never
በጭራሽ</td><td>Almost never
ብዙ አይሰጡም</td><td>Occasionally
አልፎ አልፎ</td><td>Usually
ብዙውን ጊዜ</td><td>Almost always
ሁልጊዜ ማለት ይቻላል</td><td>ሁልጊዜ</td></tr>
<tr><td colspan="6">3. Treating you as an individual.
3. ነርሷ/ነርሱ እንደሰው የሚገባዎን እንክብካቤ ይሰጡዎታል?</td></tr>
<tr><td>Never
በጭራሽ</td><td>Almost never
ብዙ አይደሉም</td><td>Occasionally
አልፎ አልፎ</td><td>Usually
ብዙውን ጊዜ</td><td>Almost always
ሁልጊዜ ማለት ይቻላል</td><td>ሁልጊዜ</td></tr>
<tr><td colspan="6">4. Spending time with you.
4. ነርሷ/ነርሱ ከእርስዎ ጋር በቂ ጊዜ ያሳልፋሉ?</td></tr>
<tr><td>Never
በጭራሽ</td><td>Almost never
በቂ ጊዜ አያሳልፉም</td><td>Occasionally
አልፎ አልፎ</td><td>Usually
ብዙውን ጊዜ</td><td>ሁልጊዜ ማለት ይቻላል</td><td>ሁልጊዜ</td></tr>
<tr><td colspan="6">5. Supporting you.
5. ነርሷ/ነርሱ አስፈላጊውን የህክምና ድጋፍ ያደርጉልዎታል?</td></tr>
<tr><td>Never
በጭራሽ</td><td>Almost never
ብዙ ድጋፍ አያደርጉም</td><td>Occasionally
አልፎ አልፎ</td><td>Usually
ብዙውን ጊዜ</td><td>ሁልጊዜ ማለት ይቻላል</td><td>ሁልጊዜ</td></tr>
<tr><td colspan="6">6. Being empathetic or identifying with you.
6. ነርሷ/ነርሱ ሩህሩህ ናቸው፤ እንደእርስዎ ሆነው ስሜትዎንና ችግርዎን ይረዱልዎታል?</td></tr>
<tr><td>Never
በጭራሽ</td><td>Almost never
ብዙ አይደሉም</td><td>Occasionally
አልፎ አልፎ</td><td>Usually
ብዙውን ጊዜ</td><td>ሁልጊዜ ማለት ይቻላል</td><td>ሁልጊዜ</td></tr>
<tr><td colspan="6">7. Being confident with you.
7. ነርሷ/ነርሱ በእርስዎ ላይ እምነት አላቸው።</td></tr>
<tr><td>Never
በጭራሽ</td><td>Almost never
ብዙ የላቸውም</td><td>Occasionally
አልፎ አልፎ</td><td>Usually
ብዙውን ጊዜ</td><td>ሁልጊዜ ማለት ይቻላል</td><td>ሁልጊዜ</td></tr>
<tr><td colspan="6">8. Demonstrating professional knowledge and skill.
8. ነርሷ/ነርሱ ሙያዊ ዕውቀትና ክህሎት ያሳያሉ።</td></tr>
<tr><td>Never
በጭራሽ</td><td>Almost never
ብዙ ክህሎት አያሳዩም</td><td>Occasionally
አልፎ አልፎ</td><td>Usually
ብዙውን ጊዜ</td><td>ሁልጊዜ ማለት ይቻላል</td><td>ሁልጊዜ</td></tr>
<tr><td colspan="6">9. Including you in planning your care.
9. ነርሷ/ነርሱ የሚያስፈልግዎን እንክብካቤ አብረው እንዲያቅዱ ያደርጋሉ።</td></tr>
<tr><td>Never
በጭራሽ</td><td>Almost never
ብዙ አያደርጉም</td><td>Occasionally
አልፎ አልፎ</td><td>Usually
ብዙውን ጊዜ</td><td>ሁልጊዜ ማለት ይቻላል</td><td>ሁልጊዜ</td></tr>
</table>

10. Treating your information confidentially.<br>10. ነርሷ/ነርሱ የታካሚዎችን መረጃ በሚስጥር ይይዛሉ።					
Never<br>በጭራሽ	*Almost never*<br>ብዙ ሚስጥር አይዙም	*Occasionally*<br>አልፎ አልፎ	*Usually*<br>ብዙውን ጊዜ	ሁልጊዜ ማለት ይቻላል	ሁልጊዜ
11. Returning to you voluntarily.<br>11. ነርሷ/ነርሱ ወደ እርስዎ በፈቃደኝነት ይመለሳሉ።					
Never<br>በጭራሽ	*Almost never*<br>ብዙ አይመለሱም	*Occasionally*<br>አልፎ አልፎ	*Usually*<br>ብዙውን ጊዜ	ሁልጊዜ ማለት ይቻላል	ሁልጊዜ
12. Talking with you.<br>12. ነርሷ/ነርሱ ከእርስዎ ጋር ያወራሉ።					
Never<br>በጭራሽ	*Almost never*<br>ብዙ አያወሩም	*Occasionally*<br>አልፎ አልፎ	*Usually*<br>ብዙውን ጊዜ	ሁልጊዜ ማለት ይቻላል	ሁልጊዜ
13. Meeting your stated and unstated needs.<br>13. ነርሷ/ነርሱ የተለያዩ ፍላጎቶችዎን ያሟሉልዎታል።					
Never<br>በጭራሽ	*Almost never*<br>ብዙ አያሟሉልኝም	*Occasionally*<br>አልፎ አልፎ	*Usually*<br>ብዙውን ጊዜ	ሁልጊዜ ማለት ይቻላል	ሁልጊዜ
14. Responding quickly when you call.<br>14. ነርሷ/ነርሱ ለጥሪዎ በፍጥነት ምላሽ ይሰጣሉ።					
Never<br>በጭራሽ	*Almost never*<br>ብዙ ምላሽ አይሰጡም	*Occasionally*<br>አልፎ አልፎ	*Usually*<br>ብዙውን ጊዜ	ሁልጊዜ ማለት ይቻላል	ሁልጊዜ
15. Giving your treatments and medications on time.<br>15. ነርሷ/ነርሱ መድኃኒቶችና ሌሎች ሕክምናዎችን በሰዓቱ ይሰጡዎታል።					
Never<br>በጭራሽ	*Almost never*<br>ብዙ ጊዜ ሰዓት አይጠብቁም	*Occasionally*<br>አልፎ አልፎ	*Usually*<br>ብዙውን ጊዜ	ሁልጊዜ ማለት ይቻላል	ሁልጊዜ
16. Relieving your symptoms.<br>16. ነርሷ/ነርሱ የሕመም ምልክቶችዎን ለማስታገስ ይጥራሉ።					
Never<br>በጭራሽ	*Almost never*<br>ብዙ አይጥሩም	*Occasionally*<br>አልፎ አልፎ	*Usually*<br>ብዙውን ጊዜ	ሁልጊዜ ማለት ይቻላል	ሁልጊዜ

Thanks for your participation!
ለተሳትፎዎ እናመሰግናለን!
